# Variant Sciatic Nerve Anatomy in Relation to the Piriformis Muscle on Magnetic Resonance Neurography: A Potential Etiology for Extraspinal Sciatica

**DOI:** 10.3390/tomography9020039

**Published:** 2023-02-22

**Authors:** Upasana Upadhyay Bharadwaj, Vanja Varenika, William Carson, Javier Villanueva-Meyer, Simon Ammanuel, Matthew Bucknor, Nathaniel M. Robbins, Vanja Douglas, Cynthia T. Chin

**Affiliations:** 1 Department of Radiology and Biomedical Imaging, University of California San Francisco, San Francisco, CA 94143, USA; 2 RadNet Northern California, RadNet Imaging Centers, San Francisco, CA 90815, USA; 3 Southwest Medical Imaging, Scottsdale, AZ 85258, USA; 4 Department of Neurological Surgery, University of Wisconsin School of Medicine and Public Health, Madison, WI 53705, USA; 5 Department of Neurology, Geisel School of Medicine at Dartmouth, Hanover, NH 03755, USA; 6 Department of Neurology, UCSF Weill Institute for Neurosciences, San Francisco, CA 94143, USA

**Keywords:** MRI, neurogram, sciatic nerve, piriformis, variant anatomy

## Abstract

Objective: To assess the prevalence and clinical implications of variant sciatic nerve anatomy in relation to the piriformis muscle on magnetic resonance neurography (MRN), in patients with lumbosacral neuropathic symptoms. Materials and Methods: In this retrospective single-center study, 254 sciatic nerves, from 127 patients with clinical and imaging findings compatible with extra-spinal sciatica on MRN between 2003 and 2013, were evaluated for the presence and type of variant sciatic nerves, split sciatic nerve, abnormal T2-signal hyperintensity, asymmetric piriformis size and increased nerve caliber, and summarized using descriptive statistics. Two-tailed chi-square tests were performed to compare the anatomical variant type and clinical symptoms between imaging and clinical characteristics. Results: Sixty-four variant sciatic nerves were identified with an equal number of right and left variants. Bilateral variants were noted in 15 cases. Abnormal T2-signal hyperintensity was seen significantly more often in variant compared to conventional anatomy (40/64 vs. 82/190; *p* = 0.01). A sciatic nerve split was seen significantly more often in variant compared to conventional anatomy (56/64 vs. 20/190; *p* < 0.0001). Increased nerve caliber, abnormal T2-signal hyperintensity, and asymmetric piriformis size were significantly associated with the clinically symptomatic side compared to the asymptomatic side (98:2, 98:2, and 97:3, respectively; *p* < 0.0001 for all). Clinical symptoms were correlated with variant compared to conventional sciatic nerve anatomy (64% vs. 46%; *p* = 0.01). Conclusion: Variant sciatic nerve anatomy, in relation to the piriformis muscle, is frequently identified with MRN and is more likely to be associated with nerve signal changes and symptomatology.

## 1. Introduction

The clinical syndrome of buttock and leg pain, known as sciatica, is an extremely common condition, with a reported lifetime prevalence of nearly 43% [1]. A discogenic etiology is the most common cause, accounting for up to 85% of sciatica cases [2]. Other causes are less common and include tumors, inflammation, vascular lesions, endometriosis, fibrosis, and piriformis syndrome [2].

Extraspinal sciatica or piriformis syndrome refer to the compression of the sciatic nerve near the piriformis muscle, and account for 6–8% of sciatica cases [3]. The pathoetiology is not well-understood, but is thought to reflect an entrapment neuropathy near the greater sciatic notch, which can result from scarring in the setting of prior gluteal trauma, muscular hypertrophy or inflammation, compressive masses, or anatomical variations in the course of the sciatic nerve in relation to the piriformis muscle [4].

**Figure 1:**
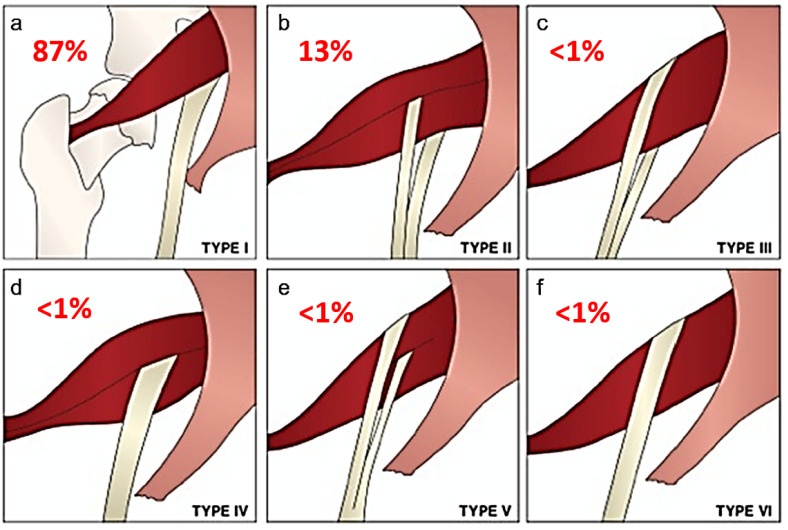
Beaton and Anson classification of different anatomical relationships between the sciatic nerve and piriformis muscle and their estimated prevalence. (**a**) Type 1: undivided sciatic nerve passing anterior and below the piriformis. (**b**) Type 2: common peroneal nerve component piercing a bifid piriformis, tibial component running in normal position anterior and inferior to piriformis. (**c**) Type 3: one division posterior to and the other anterior to the piriformis. (**d**) Type 4: undivided sciatic nerve piercing bifid piriformis. (**e**) Type 5: one division through and the other posterior to the piriformis. (**f**) Type 6: undivided nerve posterior to piriformis. Figure adapted with permission from Varenika et al. [17].

**Figure 2:**
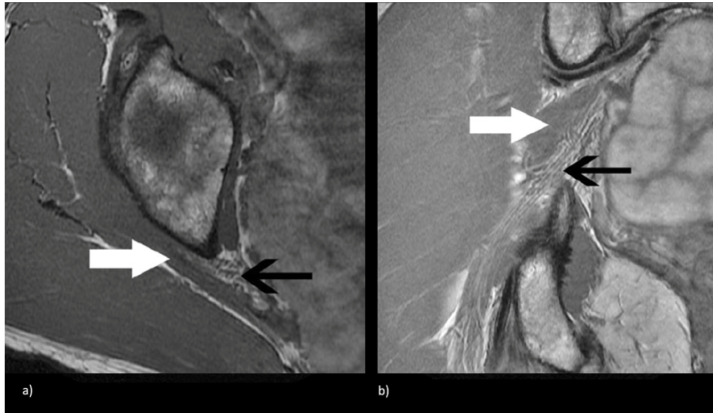
Magnetic resonance neurogram (MRN) of non-split sciatic nerve (type I). (**a**) Axial T1-weighted MRN of the sciatic nerve at the level of the sciatic notch demonstrates normal sciatic nerve (black arrow) anterior to the piriformis (white arrow). (**b**) Coronal T1-weighted sequence showing type I sciatic nerve (black arrow) inferior to the piriformis (white arrow).

**Figure 3:**
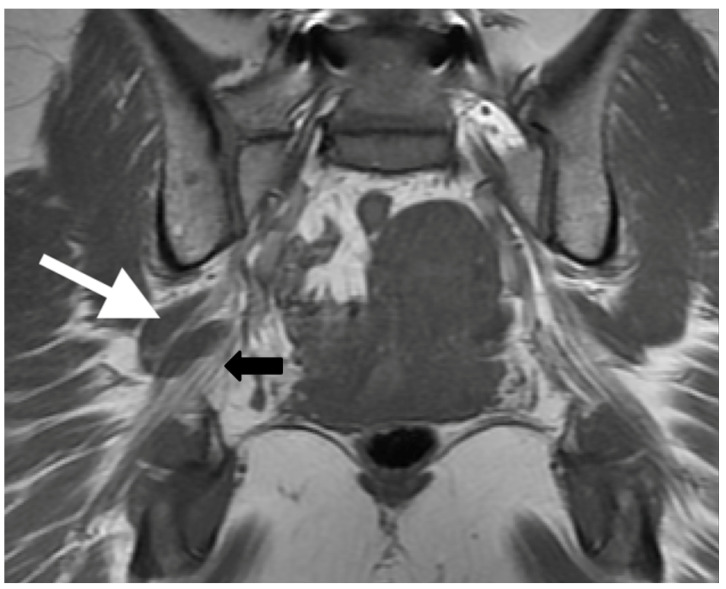
Coronal T1-weighted sequence of the bilateral sciatic nerves showing a type II variant with a common peroneal nerve component piercing a bifid piriformis (white arrow), tibial nerve component running in normal position anterior and inferior to piriformis (black arrow).

Beaton and Anson originally described six anatomical relationships between the sciatic nerve and piriformis muscle, as shown in Figure 1 [5]. Type I describes the conventional and most common relationship, in which an undivided sciatic nerve courses anterior and inferior to the piriformis muscle, visualized in Figure 2. Cadaveric studies demonstrate the prevalence of type I nerves to be approximately 87% [6,7]. The remaining 13% display variant anatomy, with the overwhelming majority of these characterized by the common peroneal nerve component running through a bifid piriformis muscle, and the tibial component remaining in the conventional location, inferior to the piriformis (type II) [6], as shown in Figure 3. The remaining variants (type III–VI) are rare, occurring in fewer than 1% of all cases.

Magnetic resonance neurography is an imaging technique that increases the imaging conspicuity of nerves by suppressing the signal from adjacent tissue [8,9]. T2-weighted sequences with fat saturation are sensitive to increased water content, hence appropriate for imaging nerve roots and peripheral nerves [10]. MR neurography has demonstrated focal signal abnormalities within the sciatic nerve in the vicinity of the sciatic notch, in patients with extraspinal sciatica [11,12,13].

Although previous cadaveric [6] and retrospective MR imaging studies [7,11,12,13,14,15,16,17] have defined the prevalence of these variant anatomical relationships between the sciatic nerve and piriformis, few studies, to our knowledge, have evaluated whether variant sciatic nerve anatomy is associated with symptomatic presentation. This study describes the imaging prevalence and clinical implications of sciatic nerve variations in patients with lumbosacral neuropathic symptoms, who underwent conventional MR neurography for evaluation of extraspinal sciatica or piriformis syndrome.

## 2. Materials and Methods

Institutional Review Board approval was obtained to perform a retrospective analysis of patients with lumbosacral plexopathy, who underwent lumbosacral plexus MR neurography from 2003 to 2013, and had clinical and imaging findings compatible with extra-spinal sciatica or piriformis syndrome.

### 2.1. Patient Cohort

Reports and findings of 1290 MR neurograms of the lumbosacral plexus from 1179 patients, acquired between February 2003 and December 2013, were extracted, including data on the ordering physician, patient demographics, clinical indications, and radiographic findings from our institution’s radiology database. Clinical information from the electronic medical records were reviewed for diagnosis of extraspinal sciatica or piriformis syndrome. Our institution’s MRN protocol was modified after the above timeframe with the addition of advanced diffusion tensor imaging sequences, which would not be available at all imaging centers; hence, we focus on conventional sequences in this study.

Patients were evaluated by board-certified neurologists, neurosurgeons, orthopedic surgeons, or pain management physicians, and were diagnosed with extraspinal sciatica based on their symptoms and examination findings, in conjunction with the absence of correlative findings on conventional imaging studies. These patients were then referred for MR neurography studies, tailored to the lumbosacral plexus, in order to identify abnormalities not yet explained by conventional imaging or other diagnostic techniques.

A total of 127 symptomatic patients underwent MR neurography imaging for extraspinal sciatica between 2003 and 2013 (254 sciatic nerves: 127 × 2). Patients with history of trauma, underlying inflammatory conditions, infections, tumors, chemoradiation, and prior history of lumbar surgery or degenerative disc disease resulting in either moderate or severe spinal stenosis, were excluded. Symptoms included numbness, weakness, or pain in the buttock, back, thigh, or foot. While all patients were symptomatic, the specific side associated with the sciatic nerve variant may not be symptomatic. Patient demographics, including age, gender, clinical symptoms, symptoms duration, electromyography abnormalities, whether the patient received neurolytic surgery and eventual clinical improvement, were extracted from clinical notes.

### 2.2. Image Acquisition

Lumbosacral plexus MR neurograms of all patients (*n* = 127) was performed as per the standard imaging protocols within our institution, using the following sequences: 2D T1-weighted spin-echo axial and coronal sequences; 2D T2-weighted fat-saturated fast spin-echo iterative decomposition of water and fat with echo asymmetry and least-squares estimation (IDEAL) axial and coronal sequences. Sequences were acquired on a Discovery MR750 scanner (GE Healthcare, Milwaukee, WI, USA) and phased-array body/torso coil (GE Healthcare, Milwaukee, WI, USA) at 1.5 Tesla (*n* = 71) and 3.0 Tesla (*n* = 56). The imaging protocols for both scanners were identical and are presented in Table 1.

### 2.3. Image Evaluation

MR neurograms were evaluated on standard Picture Archiving and Communication System (PACS) workstations (Agfa, Mortsel, Belgium) independently, by a musculoskeletal radiology fellow and an attending neuroradiologist with 17 years of experience, blinded to the clinical history and side of symptoms. The anatomical relationship between the sciatic nerve and the piriformis muscle, just distal to the greater sciatic notch, were analyzed and categorized according to the Beaton and Anson classification system [5]. Additionally, the presence of a split sciatic nerve, defined as discrete separation of the common peroneal and tibial nerve bundles by a fat plane (of any thickness) at the level of the ischial tuberosity, was recorded. Nerve caliber and T2 signal were evaluated qualitatively, at the level of the sciatic notch relative to the proximal spinal nerves in the pelvis, and sciatic nerve distal to the ischial tuberosity, with no quantitative measurements recorded; hence, no thresholds or cut-offs were employed. Similar methodology was employed in prior work describing MR neurography findings in patients with extraspinal sciatica [11]. Piriformis muscle size and morphology were also evaluated qualitatively for relative asymmetry. In cases of discrepancy (*n* = 8), a third reader, a musculoskeletal radiologist attending, blinded to the clinical history and the prior readers’ radiologic evaluation, assessed the imaging.

### 2.4. Statistical Analysis

The prevalence and type of variant sciatic nerves, presence of a split sciatic nerve, abnormal T2-signal hyperintensity, asymmetric piriformis size, and increased nerve caliber were summarized using descriptive statistics. Two-tailed chi-square tests were performed to compare anatomical variant type and clinical symptoms between imaging and clinical characteristics. Multilevel logistic regression models were used to assess predictors of variant type, as well as predictors for symptoms, after controlling for any baseline factor that was significant (*p* < 0.05) on univariate analysis. *p*-values were 2-tailed, with an alpha value of 0.05 considered statistically significant. Statistical analysis was performed using the Python SciPy v1.0 statistics module [18].

## 3. Results

### 3.1. Patient Demographics

One hundred and forty-nine patients underwent MR neurography for a clinical diagnosis of extraspinal sciatica or piriformis syndrome between February 2003 and December 2013. Of these, 16 were excluded due to a history of prior lumbar surgery or degenerative changes resulting in moderate or severe stenosis. An additional six were repeat examinations, and therefore excluded from analysis. Of the 127 included patients, 80 were women and 47 were men. The median age at time of examination was 50 years (range: 23–91, interquartile range: 18.75). At presentation, 54.0% (*n* = 68) had right-sided symptoms and 46.0% (*n* = 59) had left-sided symptoms.

### 3.2. MR Neurography of the Sciatic Nerves

Seventy-one cases (56.0%) were acquired at 1.5 tesla and 56 cases (44.0%) were acquired at 3.0 tesla; statistical significance of the difference between the two scanners across demographics and pertinent study attributes are summarized in Table 2. No significant differences were observed between the two scanners. As each sciatic nerve was considered independently, a total of 254 sciatic nerves were evaluated. Sixty-four (25.2%) variant sciatic nerves were identified with an equal number of right and left variants (32 variants each). Bilateral variants were noted in 15 cases. Of the 64 variant sciatic nerves, 63 were type II variants and one was a type III variant. No other sciatic nerve variants were identified in the study cohort.

Abnormal T2-signal hyperintensity was seen significantly more often in variant compared to conventional anatomy (40/64 vs. 82/190; *p* = 0.01). A sciatic nerve split at the level of the ischial tuberosity was also seen significantly more often in variant compared to conventional anatomy (56/64 vs. 20/190; *p* < 0.0001). The sciatic nerve type and correlation with clinical and imaging characteristics, along with results of the chi-square tests are reported in Table 3. Multilevel logistic regression showed that T2 nerve signal hyperintensity and a sciatic nerve split at the at ischial tuberosity were significant predictors of variant nerve anatomy. These results are summarized in Table 4.

### 3.3. Sciatic Nerve Correlation with Findings at MR Neurography

Increased nerve caliber, abnormal T2-signal hyperintensity, and asymmetric piriformis size were significantly associated with the clinically symptomatic side, compared to the asymptomatic side (98.0% vs. 2.0%, 98.0% vs. 2.0%, and 97.0% vs. 3.0%, respectively; *p* < 0.0001 for all).

### 3.4. Sciatic Nerve Correlation with Clinical Presentation and Outcomes

In the study cohort, 29.0% of the patients presented with buttock or low-back pain; 35.0% of the patients presented with buttock or lower-back pain, along with lower-extremity pain; 33.0% presented with lower-extremity pain, numbness, or weakness; and 3.0% presented with groin pain. Thirty-five percent of all patients underwent electrodiagnostic studies.

Clinical symptoms were more often correlated with variant compared to conventional sciatic nerve anatomy (64.0% vs. 46.0%; *p* = 0.01). No significant difference was seen in electromyography studies between conventional compared to variant sciatic nerve anatomy. Neurolytic surgery was performed at similar rates between patients with variant and conventional sciatic nerve anatomy (10.0% vs. 8.0%; *p* = 0.75). Eventual clinical improvement was also noted at similar rates between patients with variant and conventional sciatic nerve anatomy (63.0% vs. 56.0%; *p* = 0.82). Sciatic nerve type and correlation with clinical presentation and outcomes is presented in Table 5.

### 3.5. Statistical Analysis

At multivariate regression, ipsilateral, abnormal T2-signal hyperintensity and asymmetric piriformis size were found to be predictors of symptoms (*p* < 0.0001 and *p* = 0.002, respectively) with odds ratios of 1321.53 (95% CI: (150.96, 11,569.27)) and 57.64 (95% CI: (4.35, 763.03)), respectively, as shown in Table 6. Significant associations were not observed for variant sciatic nerve anatomy or sciatic nerve enlargement.

## 4. Discussion

**Figure 4:**
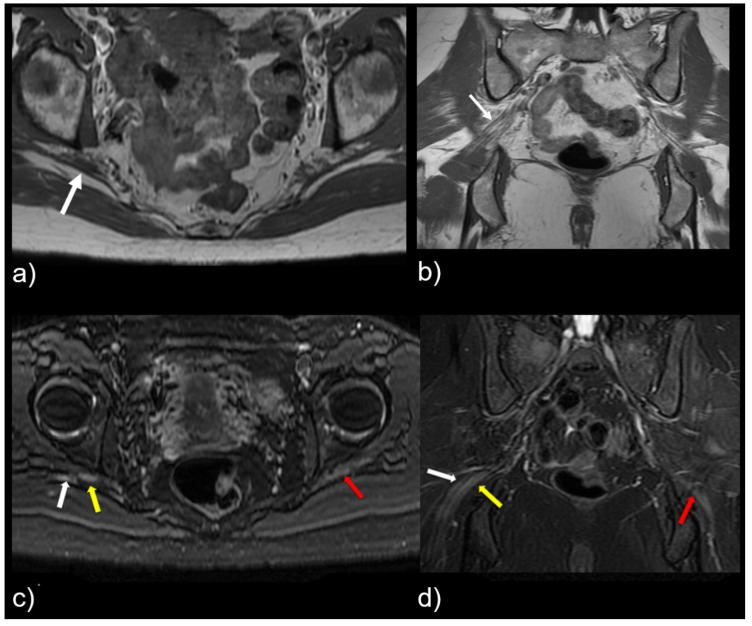
Patient with a symptomatic type II variant and a split right sciatic nerve. Axial (**a**) coronal (**b**) T1-weighted sequence, showing the two components of the split sciatic nerve (white arrow). Axial (**c**) and coronal (**d**) T2-weighted IDEAL sequences demonstrate the split sciatic nerve, with the common peroneal (white arrow) and the tibial (yellow arrow) nerve components seen distinctively. There is also increased nerve caliber and increased nerve T2 signal involving the split right sciatic nerve, relative to the normal caliber and T2 signal of the left sciatic nerve (red arrow).

This study shows that variant sciatic nerve anatomy in relation to the piriformis muscle can be identified by MR neurography, and is more likely to be associated with nerve signal changes and corresponding symptomatology. R egardless of the variant sciatic nerve type, imaging findings that strongly correlated with the symptomatic side are abnormal nerve T2 signal, increased nerve caliber, and asymmetric piriformis size (Figure 4). Variant sciatic nerve anatomy may occur in up to 13% of the population [6,17] and can be difficult to identify without advanced imaging, such as MR neurography. Variant sciatic nerve anatomy may therefore represent an under-diagnosed cause of extraspinal sciatica and piriformis syndrome. To our knowledge, prior studies have not evaluated the imaging prevalence of these variants on MR neurography and their clinical implications.

The study cohort had a median age of 50, consistent with the peak prevalence of sciatica occurring in the fourth and fifth decades of life. The population also contained almost twice as many females (*n* = 80) as males (*n* = 47), although prior large, cross-sectional studies failed to demonstrate any influence of gender on the development of sciatica [19].

Our study suggests a 25.2% prevalence of variant sciatic nerve anatomy (all but one type II variant), which is nearly double the estimated population prevalence of 13.0% [6,7]. This discrepancy can be partly explained by selection bias, in that all of patients in the cohort had a clinical diagnosis of extraspinal sciatica. In combination with the finding that the majority (64.0%) of sciatic nerve variants detected in our study were located on the symptomatic side, the increased prevalence further supports the hypothesis that variant sciatic anatomy is associated with increased symptoms.

**Figure 5:**
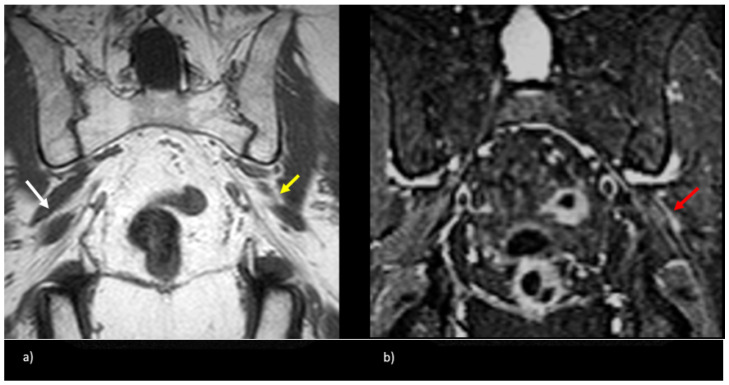
Patient presented with left-sided symptoms and lumbosacral plexus MRN showing bilateral type II variants: (**a**) coronal T1-weighted sequence showing right-sided sciatic nerve split around the normal right (white arrow) and atrophied left (yellow arrow) piriformis muscle; (**b**) coronal STIR sequence demonstrating increased signal of the left sciatic nerve (red arrow).

Variant sciatic nerve anatomy also showed a statistically significant (*p* = 0.01) increased rate of T2-signal abnormality (63.0% variant vs. 43.0% normal). In both groups, the signal abnormality occurred at, or just distal to, the greater sciatic foramen, possibly reflecting the most vulnerable site for nerve entrapment or compression. Furthermore, regardless of variant sciatic nerve type, increased nerve T2 signal strongly correlated with the symptomatic side (98.0%). Figure 5 illustrates this finding, with bilateral type II variant split sciatic nerves in a patient who presented with symptoms on the left side, wherein increased signal was demonstrated only on the left (symptomatic) side. Although only a minority of variant and normal nerves displayed an asymmetric nerve caliber or asymmetric piriformis size (hypertrophy or atrophy), both imaging findings were significantly associated with the symptomatic side (98.0%). A detailed evaluation of variant anatomy is therefore essential for an accurate diagnosis.

Sciatic nerve variants may be associated with increased symptoms due to more traction on the traversing nerves by the aberrant anatomic paths. Focal nerve T2-signal abnormality in a common consistent location in the greater sciatic foramen in symptomatic patients may, therefore, be an imaging sign of piriformis entrapment neuropathy, in line with similar findings documented in other studies of MR neurography and extraspinal sciatica [11,12,13].

Variant nerves are more likely to be associated with a split sciatic nerve compared to normal (88.0% vs. 11.0%, *p* < 0.0001). The presence of a split sciatic nerve at the ischial tuberosity warrants a careful evaluation of its more proximal portions around the piriformis muscle and the sciatic notch.

Patients with variant, as well as normal, sciatic nerve anatomy had normal electromyography findings overall, which is consistent with prior nerve conduction studies in piriformis syndrome. Since routine electromyography is typically performed in a relaxed patient position, sciatic nerve compression by the piriformis muscle may be implicitly minimized. Previous studies demonstrate a delay in the H reflex on EMG in the FAIR position (hip flexion, abduction, and internal rotation) in patients with piriformis syndrome, compared to asymptomatic controls [20,21].

Patients with normal, as well as variant, sciatic anatomy showed eventual clinical improvement with non-surgical therapies, which consisted of physical therapy and targeted piriformis injections with the botulinum toxin. Only a minority (8.0%) required surgery, including sectioning the piriformis muscle and release of any fascial bands or vessels compressing the nerve. Although our study does not conclusively associate variant anatomy with outcomes, prior studies suggest that they are favorable [13].

While advanced imaging techniques, such as diffusion tensor imaging (DTI), have been introduced for the visualization of the sciatic nerve [22], we analyze an important study cohort that did not rely on DTI or other advanced imaging techniques. Given the widespread prevalence of nerve injury, our study shows that routine clinical sequences can be used to diagnose extraspinal sciatica without expensive, specialized coils that may be inaccessible in resource-limited imaging centers.

A limitation of our study is the qualitative nature of evaluation, wherein differences in attributes, such as nerve caliber size and T2-signal intensity, were not characterized quantitatively; however, our assessment was based on the comparison of sciatic nerve caliber and T2 signal at the level of the sciatic notch relative to the proximal and distal ipsilateral, as well as the contralateral, sciatic nerves, noting relative asymmetry in line with prior studies [11]. Since the readers were discrepant in only a small subset of cases (*n* = 8), we believe this limitation likely does not obfuscate key findings of this study.

The study cohort did not contain any polyneuropathies based on clinical records and electrodiagnostic studies, and limits evaluation of the association between sciatic nerve variants and polyneuropathies, which is an interesting direction for future research on a larger cohort.

Another potential concern in the evaluation of the nerve signal is the impact of magic-angle phenomenon, leading to an artificially increased T2 signal [23]; our imaging parameters utilized an echo time (TE) of 70 ms for IDEAL MR neurography sequences, which is greater than the 66 ms that has been reported to be necessary to avoid the magic-angle phenomenon [24]. Lastly, we acknowledge that the results of this study are from a single center and may not be applicable in settings where the patient demographics or imaging protocols may be substantially different from ours.

## 5. Conclusions

Variant sciatic nerve anatomy in relation to the piriformis muscle can be associated with symptoms of extraspinal sciatica, as well as MR neurography findings that include hyperintense nerve T2 signal, relatively increased nerve caliber, and asymmetric piriformis size. Thorough evaluation of sciatic nerve variants is, therefore, suggested for a comprehensive assessment and potential diagnosis of extraspinal sciatica or piriformis syndrome.

## Figures and Tables

**Table 1:** Acquisition parameters used in this study for various magnetic resonance neurography (MRN) sequences.

MR Sequence	Orientation	Fat Saturation	FOV (cm)	Slice Thickness (mm)	TR (ms)	TE (ms)	Matrix (Pixels)	NEX
T1-weighted	Axial	No	24	3	600	min	384 × 192	3.5 × 4
T1-weighted	Coronal	No	24	3	700	min	384 × 224	5 × 4
T2-weighted IDEAL	Axial	Yes	24	3	3700	70	256 × 160	6.5 × 2
T2-weighted IDEAL	Coronal	Yes	24	3	3000	70	288 × 192	5 × 2

**Table 2:** Statistical significance of the difference between the MR images acquired at 1.5 T and 3.0 T for pertinent study attributes.

Attribute	Age	Gender	Symptoms	Surgery	Sciatic Nerve Variant	Increased Nerve T2 Signal	Split Sciatic at Ischial Tuberosity	Asymmetric Piriformis Size	Increased Nerve Caliber
*p*-value	0.79	0.48	0.62	0.63	0.72	0.1	0.94	0.07	0.93

**Table 3:** Sciatic nerve type and correlation with clinical and imaging characteristics.

	Variant (*n* = 64)	Normal (*n* = 190)	*p*-Value
Symptomatic	41/64 (64%)	87/190 (46%)	0.01
Increased nerve T2 signal	40/64 (63%)	82/190 (43%)	0.01
Split sciatic at ischial tuberosity	56/64 (88%)	20/190 (11%)	<0.0001
Asymmetric piriformis size	11/64 (17%)	25/190 (13%)	0.41
Increased nerve caliber	10/64 (16%)	33/190 (17%)	0.85

**Table 4:** Odds ratio and 95% confidence interval of imaging characteristics as predictors, and anatomical variant as outcome.

	Odds Ratio	95% Confidence Interval	*p*-Value
Symptomatic	1.74	[0.29, 10.10]	0.54
Increased nerve T2 signal	2.75	[1.45, 5.24]	0.02
Split sciatic at ischial tuberosity	131.41	[40.01, 431.56]	0.001
Asymmetric piriformis size	1.54	[0.70, 3.39]	0.38
Increased nerve caliber	0.70	[0.37, 1.30]	0.72

**Table 5:** Sciatic nerve type and correlation with clinical presentation and outcomes.

	Variant (*n* = 64)	Normal (*n* = 190)	*p*-Value
Symptomatic	64%	46%	0.01
Median symptom duration	1.50 years	1.75 years	0.23
Abnormal EMG	23%	33%	0.70
Need for surgery (neurolysis)	10%	8%	0.75
Eventual clinical improvement	63%	56%	0.82

**Table 6:** Odds ratio and 95% confidence interval of imaging characteristics as predictors, and symptoms as outcome.

	Odds Ratio	95% Confidence Interval	*p*-Value
Anatomical variant	1.74	[0.29, 10.10]	0.54
T2 hyperintense signal	1321.53	[150.96, 11,569.27]	<0.0001
Nerve caliber size	0.57	[0.03, 9.66]	0.69
Asymmetric piriformis	57.64	[4.35, 763.03]	0.001

## Data Availability

All data pertinent to this study have been summarized in the tables and figures; original patient data cannot be released due to ethical considerations.
